# *Drosophila Gr64e* mediates fatty acid sensing via the phospholipase C pathway

**DOI:** 10.1371/journal.pgen.1007229

**Published:** 2018-02-08

**Authors:** Hyeyon Kim, Haein Kim, Jae Young Kwon, Jeong Taeg Seo, Dong Min Shin, Seok Jun Moon

**Affiliations:** 1 Department of Oral Biology, BK21 PLUS, Yonsei University College of Dentistry, Yonsei-ro 50–1, Seodaemun-gu, Seoul, Korea; 2 Department of Biological Sciences, Sungkyunkwan University, Suwon, Gyeonggi-do, Korea; The University of North Carolina at Chapel Hill, UNITED STATES

## Abstract

Animals use taste to sample and ingest essential nutrients for survival. Free fatty acids (FAs) are energy-rich nutrients that contribute to various cellular functions. Recent evidence suggests FAs are detected through the gustatory system to promote feeding. In *Drosophila*, phospholipase C (PLC) signaling in sweet-sensing cells is required for FA detection but other signaling molecules are unknown. Here, we show *Gr64e* is required for the behavioral and electrophysiological responses to FAs. GR64e and TRPA1 are interchangeable when they act downstream of PLC: TRPA1 can substitute for GR64e in FA but not glycerol sensing, and GR64e can substitute for TRPA1 in aristolochic acid but not N-methylmaleimide sensing. In contrast to its role in FA sensing, GR64e functions as a ligand-gated ion channel for glycerol detection. Our results identify a novel FA transduction molecule and reveal that *Drosophila Grs* can act via distinct molecular mechanisms depending on context.

## Author summary

Fatty acids (FAs) are energy-rich nutrients that are detected through the gustatory system to promote feeding. Here, we show FA detection requires a *Drosophila* gustatory receptor, *Gr64e*. Although GR64e functions as a ligand-gated ion channel for glycerol detection, in FA sensing, it acts downstream of phospholipase C signaling. We identified a novel signaling molecule for FA sensing in *Drosophila*. Furthermore, our findings suggest *Drosophila* GRs have multiple modes of action depending on their cellular and molecular context.

## Introduction

Animals use gustatory systems to evaluate the quality of food. Gustation is essential not only to prevent ingestion of toxic chemicals but also to ensure ingestion of essential nutrients such as sugars, amino acids, and lipids. The detection and consumption of energy-dense foods can confer a survival advantage, especially when food is scarce. Lipids are more calorie-rich than proteins or sugars, so it is unsurprising that lipid sensing has emerged as a new candidate taste modality in addition to the five basic taste modalities in mammals: sweet, umami, bitter, sour, and salt. Dietary lipid sensing was thought to be mediated by texture and olfaction [1–3], but the recently discovered taste receptors for fatty acids (FAs) in mammals indicate gustatory systems can also detect lipids [4, 5]. Two G-protein coupled receptors (GPCRs), GPR40 and GPR120, are present in the taste receptor cells of mammals [5, 6] and are partly requried for FA preference [5]. FA-induced responses depend on phospholipase C (PLC) and its downstream signaling molecules like transient receptor potential channel type M5 (TRPM5) [7], suggesting that FA taste is mediated by a phosphoinositide-based signaling pathway.

*Drosophila melanogaster* can detect several taste modalities including sweet, bitter, salt, and amino acids [8, 9]. Most taste modalities are detected by the direct activation of ion channels expressed in gustatory receptor neurons (GRNs). The 68 members of the gustatory receptor (*Gr*) gene family in the *Drosophila* genome include the main taste receptors for the sweet and bitter modalities [10, 11]. Although GRs have seven transmembrane domains, these proteins are not GPCRs. They have an opposite membrane topology [12, 13] and function as ligand-gated ion channels [14, 15]. Ionotropic receptors (Irs), which are distantly related to ionotropic glutamate receptors [16], are involved in the detection of low salt, pheromones, polyamines, and amino acids [17–20].

In contrast to other taste modalities, *Drosophila* FA taste signaling is mediated by the PLC pathway [21]. Mutation of *norpA*, a *Drosophila* orthologue of PLC, results in reduced attraction to FAs. The introduction of a *norpA* cDNA into sweet GRNs of *norpA*^*P24*^ flies rescues their deficit in FA sensing, suggesting PLC in sweet GRNs is essential for FA sensing. FA detection requires PLC signaling in sweet GRNs, but no other signaling molecules have yet been implicated. Here, we show that *Gr64e*, which is known as a glycerol receptor [22], is required downstream of PLC for the detection of FAs. The precise deletion of the *Gr64* cluster via CRISPR/Cas9 reduces FA palatability. By screening individual *Gr64* cluster gene mutant flies, we identified a requirement for *Gr64e* in FA sensing. We also found the re-introduction of *Gr64e* into *Gr64* cluster deletion mutants rescues their behavioral attraction to FAs and FA-evoked action potentials. *Gr64e* seems to function as a ligand-gated ion channel for glycerol sensing because the co-expression of *Gr64e* and *Gr64b* confers glycerol responses independent of PLC on sweet GRNs, the low-salt sensing GRNs, and bitter GRNs of *Gr64* cluster mutant flies. In contrast, the introduction of *TrpA1*, which can couple to PLC signaling [23, 24], in sweet GRNs of flies lacking *Gr64e* rescues their deficit in FA sensing but not glycerol sensing. In addition, *Gr64e* expression in *TrpA1* mutants can only rescue their deficit in aristolochic acid (ARI) sensing [23], which is PLC-dependent. *Gr64e* expression does not rescue the *TrpA1* mutant defect in *N*-methylmaleimide (NMM) sensing, which proceeds via direct TRPA1 activation [25]. Together, our results reveal a novel component in *Drosophila* for signal transduction in FA detection and suggest *Drosophila Grs* can function via multiple molecular mechanisms depending on their cellular and molecular context.

## Results

### The *Gr64* cluster is required for lipid sensing

**Fig 1:**
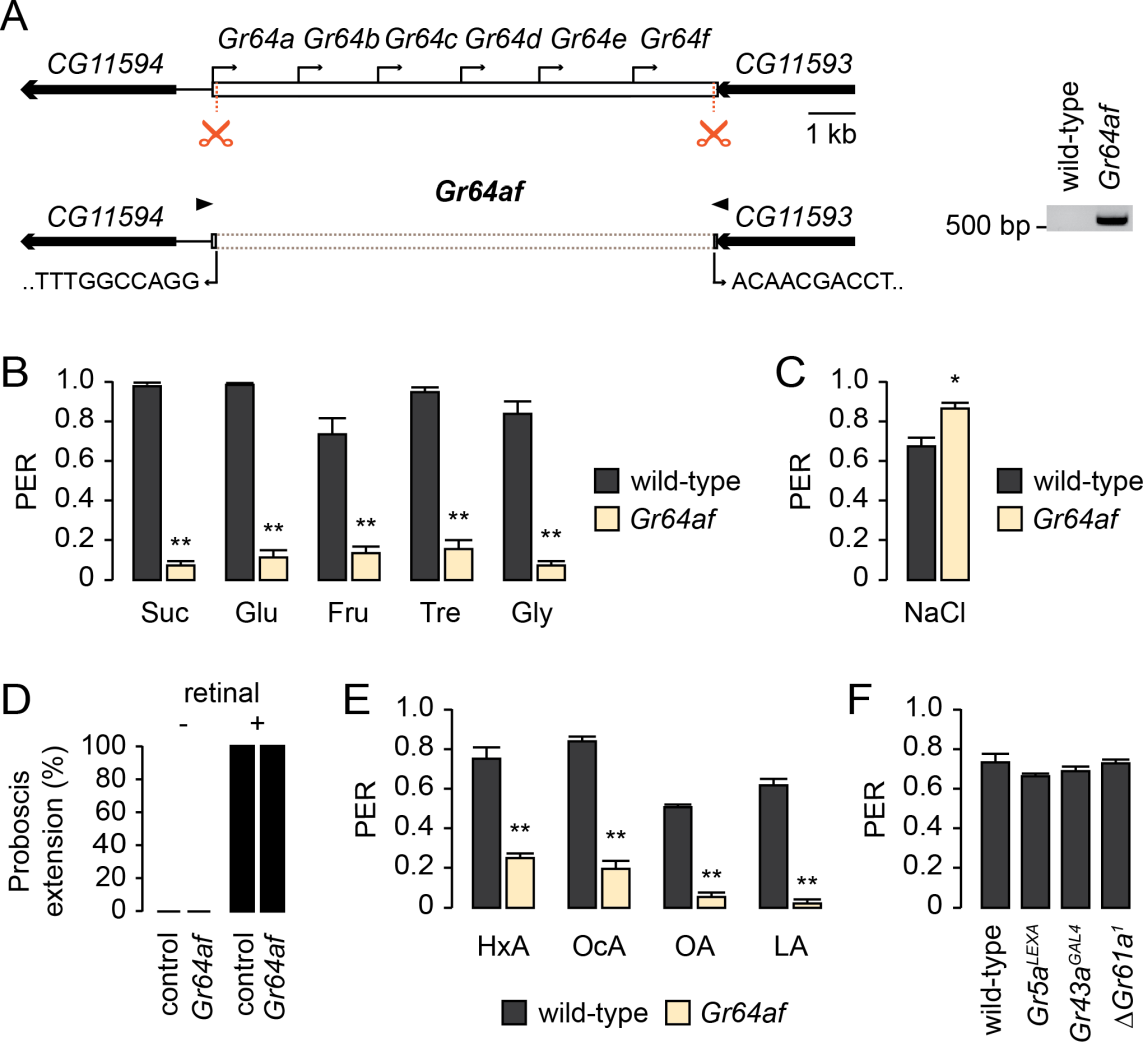
The *Gr64* cluster is required for fatty acid sensing. (A) Schematics of the *Gr64* cluster locus and the strategy for generating *Gr64af* using the CRISPR/Cas9 system. The scissors indicate the guide RNA targeting sites cut by Cas9 and the bent arrows indicate the regions where excision occurred. The arrow heads indicate the primers used for deletion validation. Genomic PCR is shown on the right. (B) Labellar PER responses to various sugars in *Gr64af* flies. 100 mM sucrose (Suc), 500 mM glucose (Glu), 100 mM fructose (Fru), 500 mM trehalose (Tre), and 5% glycerol (Gly) solutions were used. n = 9. **p < 0.001 (unpaired Student’s *t*-test). (C) Labellar PER response to low salt (50 mM NaCl) in *Gr64af* flies. n = 8. *p < 0.01 (unpaired Student’s *t*-test). (D) Optogenetic activation of sweet GRNs in two groups, *Gr5a*>*ReaChR* (control) and *Gr5a*>*ReaChR*;*Gr64af* (*Gr64af*), with retinal (+) and without retinal (-). n = 7–10. (E) Labellar PER responses to various FAs in *Gr64af* flies. 0.4% solutions of hexanoic acid (HxA), octanoic acid (OcA), oleic acid (OA), and linoleic acid (LA) were used. n = 8. **p < 0.001 (unpaired Student’s *t*-test). (F) Labellar PER responses to HxA in the indicated genotypes. A 0.4% HxA solution was used. *n* = 5–11. All data are presented as means ± SEM.

We were prompted to test whether the *Gr64* cluster is involved in FA sensing because the *Gr64* cluster is required for the detection of most phagostimulatory substances [26–31]. The *Gr64* cluster comprises six tandem *Gr* genes (*Gr64a*-*Gr64f*) transcribed as a polycistronic mRNA (Fig 1A) [26, 29, 31]. Because deletion of the whole *Gr64* cluster (*ΔGr64*) is lethal due to the additional deletion of neighboring genes [31], we used CRISPR/Cas9 to generate a new *Gr64* cluster deletion (*Gr64af*) covering only the *Gr64* cluster coding region (Fig 1A). We confirmed the deletion of the *Gr64* loci by genomic PCR and DNA sequencing (Fig 1A). In contrast to *ΔGr64*, *Gr64af* is viable and fertile. As expected, we found *Gr64af* flies show a reduced proboscis extension reflex (PER) to sucrose, glucose, fructose, trehalose, and glycerol (Fig 1B). PER responses to low salt are slightly increased compared to wild-type (Fig 1C), suggesting *Gr64af* does not have a general defect in gustatory function. Furthermore, optogenetic activation of sweet GRNs expressing red activatable channelrhodopsin (ReaChR) [32] induces PER in wild-type and *Gr64af* flies (Fig 1D), confirming that sweet GRNs of *Gr64af* are functional. We, next asked whether the *Gr64* cluster is required for FA sensing. Although wild-type flies show a robust PER response to hexanoic acid (HxA), octanoic acid (OcA), oleic acid (OA), and linoleic acid (LA), *Gr64af* flies show severely reduced PER responses to all the FAs we tested (Fig 1E). We were also able to confirm that the other sweet *Grs* (*Gr5a*, *Gr43a*, and *Gr61a*) are not required for FA sensing (Fig 1F).

### Identification of the *Gr* required for FA sensing

**Fig 2:**
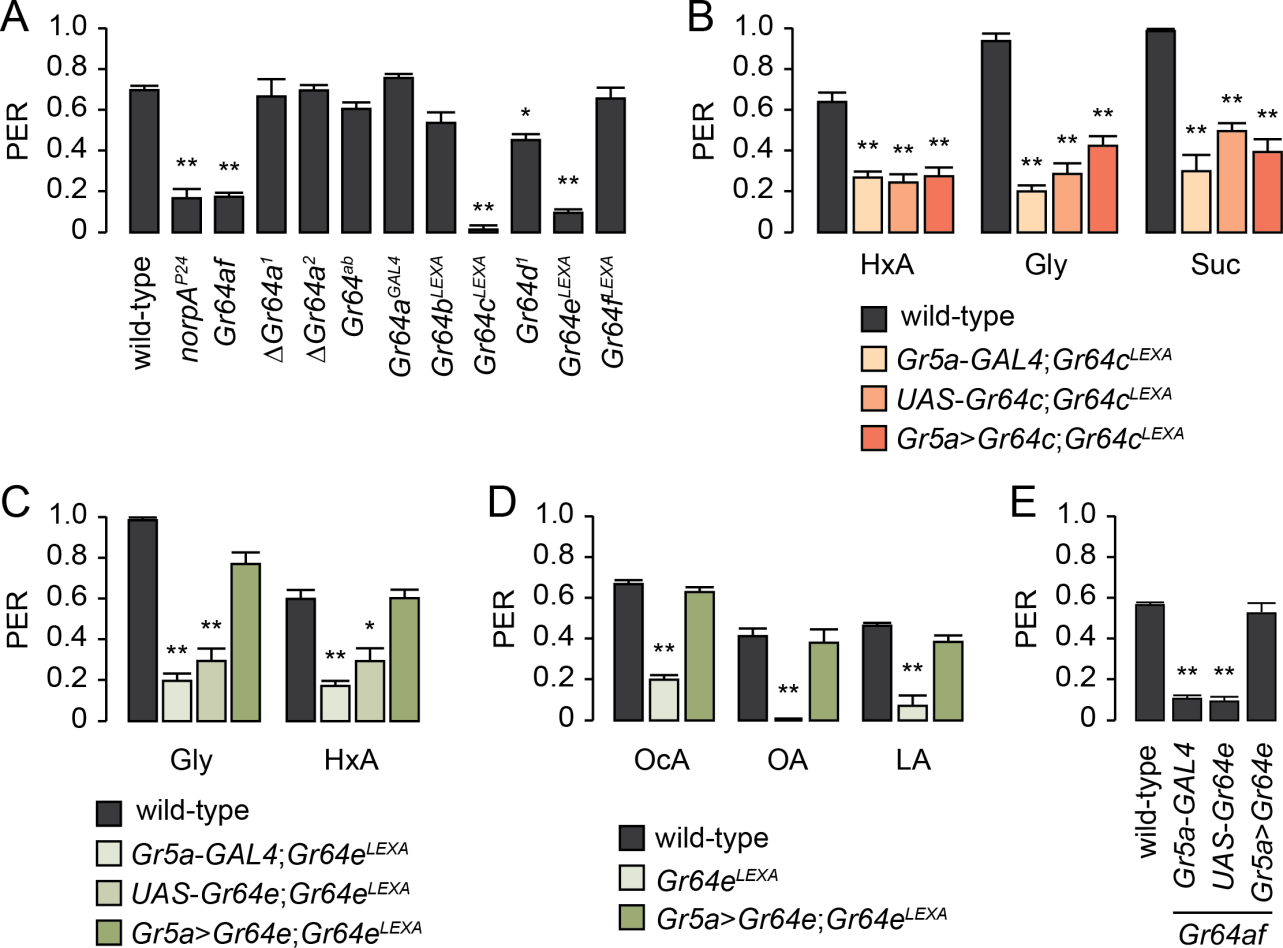
*Gr64e* is required for fatty acid sensing. (A) PER screening for individual *Gr64* cluster genes required for HxA sensing. A 0.4% HxA solution was used. *norpA*^*P24*^ was included as a positive control. n = 6–11. *p < 0.01, **p < 0.001 (one-way ANOVA with *post*-*hoc* Tukey tests). (B) Testing whether *Gr64c* is required for labellar PER responses to HxA. To test the rescue of the *Gr64c*^*LEXA*^ phenotype, we expressed a *Gr64c* cDNA in the *Gr64c*^*LEXA*^ background using *Gr5a*-*GAL4*. 0.4% HxA, 5% Gly, and 100 mM Suc solutions were used. n = 6–9. **p < 0.001 (one-way ANOVA with *post*-*hoc* Tukey tests). (C) PER analysis to determine whether *Gr64e* is required for labellar PER responses to glycerol and HxA. We expressed a *Gr64e* cDNA in *Gr64e*^*LEXA*^ flies using *Gr5a*-*GAL4*. 0.4% HxA and 5% Gly solutions were used. n = 6–10. *p < 0.01, **p < 0.001 (one-way ANOVA with *post*-*hoc* Tukey tests). (D) PER analysis to determine whether *Gr64e* is required for labellar PER responses to various FAs. 0.4% FAs were used. n = 4–8. **p < 0.001 (one-way ANOVA with *post*-*hoc* Tukey tests). (E) Rescue of the *Gr64af* defect in HxA sensing by expressing *Gr64e* under the control of *Gr5a*-*GAL4*. A 0.4% HxA solution was used. n = 6–14. **p < 0.001 (one-way ANOVA with *post*-*hoc* Tukey tests). All data are presented as means ± SEM.

To determine which of the six *Grs* in the *Gr64* cluster are required for FA sensing, we examined PER responses to HxA in flies carrying mutations in the individual genes of the *Gr64* cluster (S1 Fig). *norpA*^*P24*^ flies, which carry a mutation in the *Drosophila* orthologue of PLC [33], show reduced PER responses to HxA like *Gr64af* flies (Fig 2A) [21]. Of the various *Gr64* cluster mutants, we found *Gr64c*^*LEXA*^ and *Gr64e*^*LEXA*^ flies show reduced PER responses to HxA like the *norpA*^*P24*^ and *Gr64af* mutants (Fig 2A).

To confirm the requirement of *Gr64c* and *Gr64e* for HxA sensing, we further characterized the *Gr64c* and *Gr64e* mutants. Although *Gr64c*^*LEXA*^ flies show reduced PER responses to HxA, glycerol, and sucrose (Fig 2B), the expression of a *Gr64c* cDNA in *Gr64c*^*LEXA*^ flies using *Gr5a*-*GAL4*, which labels sweet GRNs [34], does not rescue this defect. This suggests the *Gr64c*^*LEXA*^ phenotype cannot be attributed to the loss of *Gr64c* in labellar sweet GRNs. This result is also consistent with the strong FA preference of *ΔGr64a*^*2*^ flies, which harbor a deletion of the protein-coding sequence of *Gr64a* and *Gr64b* as well as a third of the protein-coding sequence of *Gr64c* at its N-terminus (S1 Fig, Fig 2A). *Gr64e* is known as a glycerol receptor [22]. *Gr64e*^*LEXA*^ flies show reduced PER responses to glycerol and to several FAs (i.e., HxA, OcA, OA, and LA) (Fig 2C and 2D). Expression of a *Gr64e* cDNA in the *Gr64e* mutant background using *Gr5a*-*GAL4* rescues glycerol and FA responses to wild-type levels, indicating *Gr64e* is required for both glycerol and FA detection (Fig 2C and 2D). In addition, the expression of *Gr64e* using *Gr5a*-*GAL4* rescues the HxA responses of *Gr64af* flies, suggesting *Gr64e* is the only *Gr* in the *Gr64* cluster required for FA sensing (Fig 2E).

### Identifying the *Gr* that detects FA in labellar sensilla

**Fig 3:**
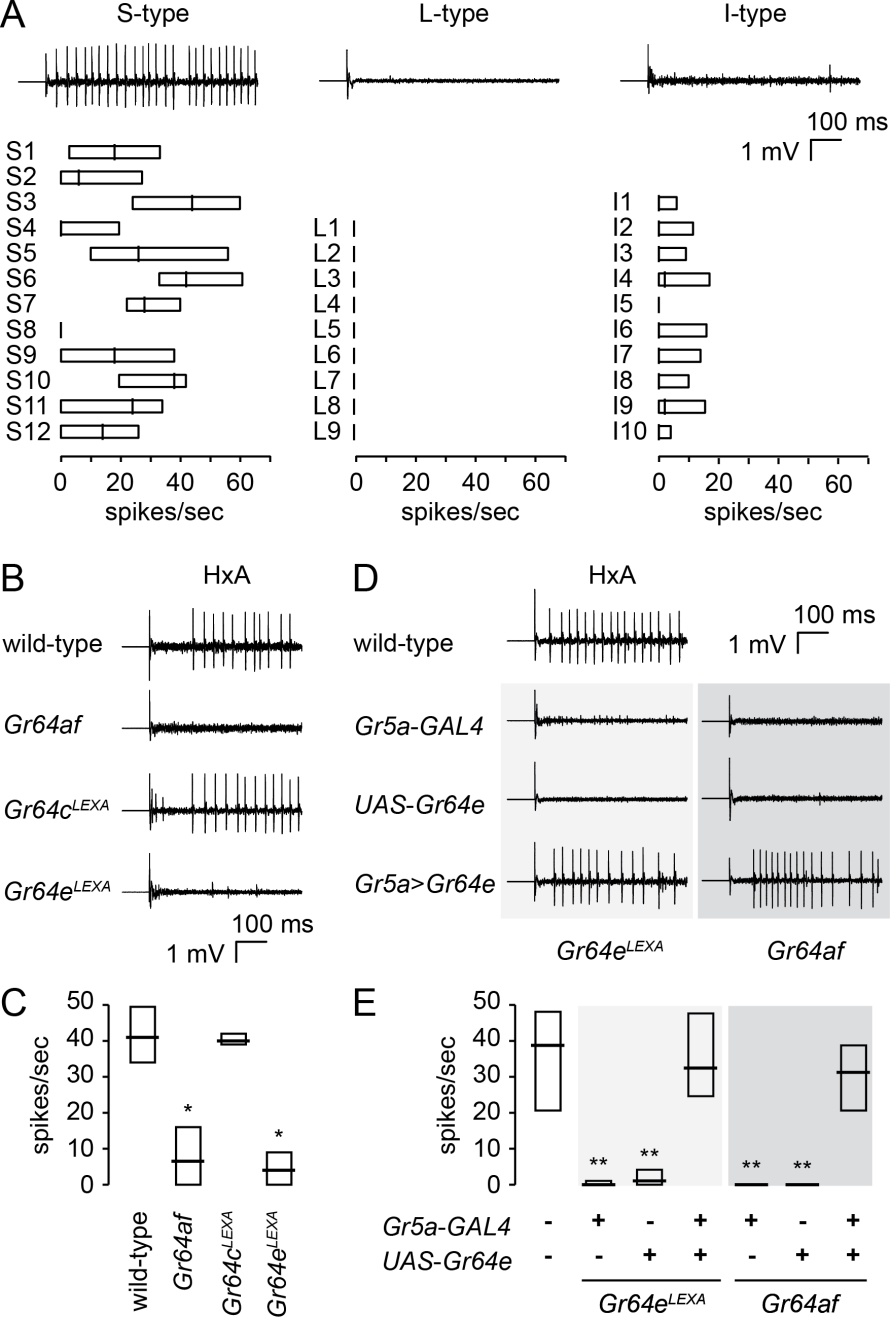
*Gr64e* is required for electrophysiological responses to HxA. (A) Electrophysiological response profiles of labellar sensilla to 1% HxA. Representative traces are shown above and action potential frequencies in the indicated sensilla are shown below. n = 3–25. (B and C) Representative traces from S6 sensilla (B) and response frequencies (C) evoked by 1% HxA in the indicated genotypes. n = 5–11. *p < 0.01 (one-way ANOVA with *post*-*hoc* Tukey tests). (D and E) Testing whether *Gr64e* is required for HxA-evoked responses. Representative traces (D) and response frequencies (E) from S6 sensilla evoked by 1% HxA. We expressed a *Gr64e* cDNA in *Gr64e*^*LEXA*^ flies or *Gr64af* flies using *Gr5a*-*GAL4*. n = 7–10. **p < 0.001 (Kruskal-Wallis with Mann-Whitney *U post-hoc* tests). Data are presented as medians with quartiles (A, C, and E).

Silencing the labellar *Gr64e*-expressing GRNs by expression of the potassium channel Kir2.1 [35] abolishes PER to HxA, suggesting that preference to HxA is mediated by *Gr64e*-expressing GRNs (S2 Fig). To better understand FA sensing in the labellum, we examined electrophysiological responses to HxA. HxA elicits action potentials mainly in S-type sensilla of wild-type flies (Fig 3A). In a few cases, we also observed HxA-evoked firing in I-type sensilla, but such responses were rare. Consistent with our PER results, we did not observe any responses to HxA in *Gr64af* or *Gr64e*^*LEXA*^ flies (Fig 3B and 3C). *Gr64c*^*LEXA*^ flies show robust, wild-type-like HxA responses, indicating that the reduced attraction of *Gr64c*^*LEXA*^ flies to HxA cannot be attributed to a peripheral defect in FA detection (Fig 3B and 3C). In addition, *Gr5a*-*GAL4*-driven expression of *Gr64e* in *Gr64e*^*LEXA*^ and *Gr64af* flies restores HxA-evoked action potentials, which suggests *Gr64e* is the only *Gr* in the *Gr64* cluster required for FA sensing (Fig 3D and 3E).

### Dual molecular functions of *Gr64e* in sweet GRNs

**Fig 4:**
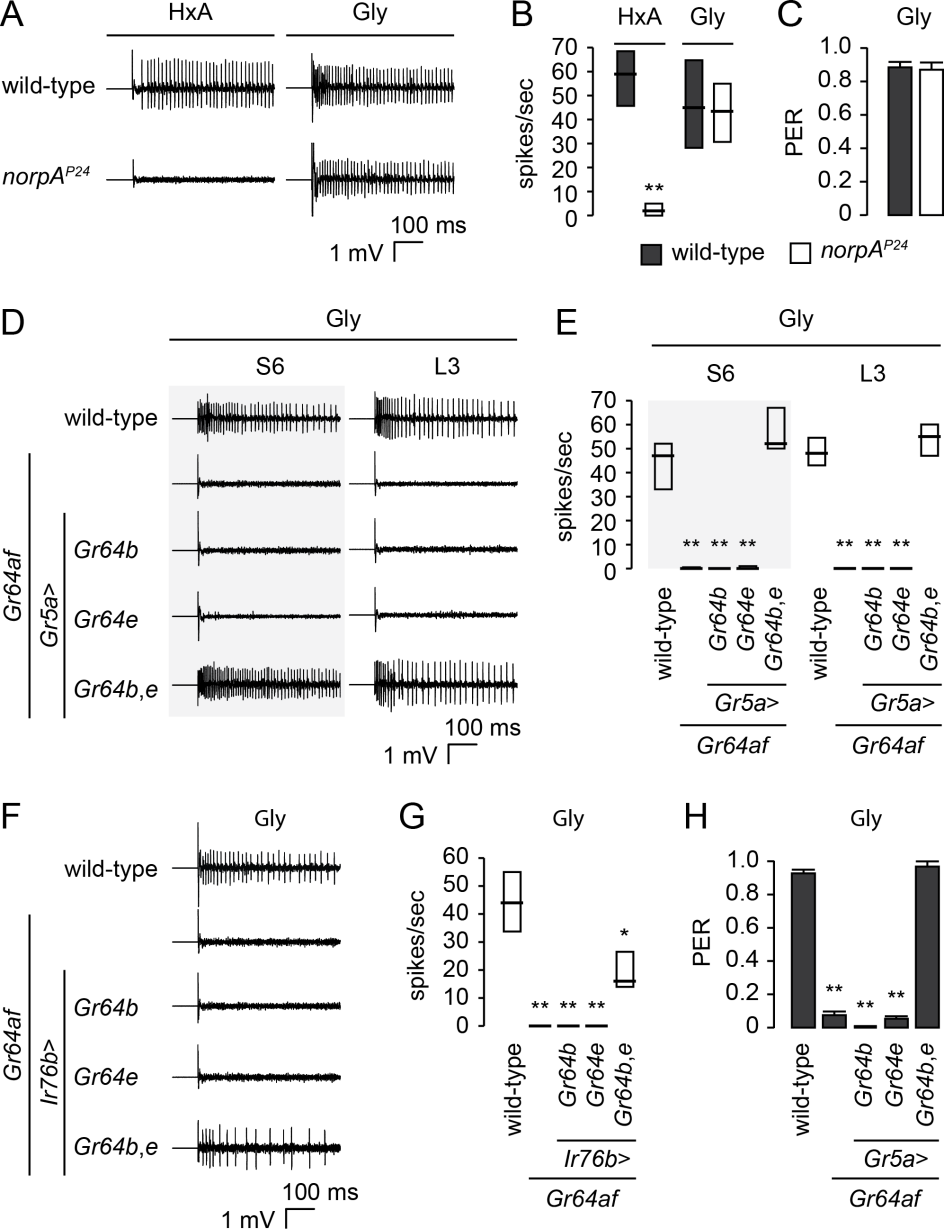
Co-expression of *Gr64b* and *Gr64e* confers glycerol responsiveness. (A and B) Representative traces (A) and response frequencies (B) from S6 sensilla in *norpA*^*P24*^ flies elicited by 1% HxA and 10% glycerol solutions. n = 5–10. **p < 0.001 (Mann-Whitney *U* test). (C) Labellar PER responses to glycerol in *norpA*^*P24*^ flies. A 5% glycerol solution was used. n = 5–7. (D and E) Representative traces (D) and response frequencies (E) from the indicated sensilla of *Gr64af* flies co-expressing *Gr64b* and *Gr64e* in sweet GRNs elicited by 10% glycerol. n = 5–13. **p < 0.001 (Kruskal-Wallis with Mann-Whitney *U post-hoc* tests). (F and G) Representative traces (F) and response frequencies (G) from L6 sensilla of *Gr64af* flies co-expressing *Gr64b* and *Gr64e* in low salt-sensing GRNs elicited by 10% glycerol. n = 4–20. *p < 0.01, **p < 0.001 (Kruskal-Wallis with Mann-Whitney *U post-hoc* tests). (H) Labellar PER responses to glycerol in *Gr64af* flies co-expressing *Gr64b* and *Gr64e* using *Gr5a*-*GAL4*. A 5% glycerol solution was used. n = 3–9. **p < 0.001 (one-way ANOVA with *post*-*hoc* Tukey tests). Data are presented as medians and quartiles (B, E, and G) or as means ± SEM (C and H).

*Gr64e* is required in GRNs for electrophysiological and behavioral responses to glycerol [22]. To determine whether the molecular function of *Gr64e* is the same in the detection of glycerol and FAs, we next asked whether PLC is required for glycerol sensing. We found no difference between wild-type and *norpA*^*P24*^ flies in glycerol-evoked action potentials or PER responses (Fig 4A–4C). This indicates *Gr64e* plays distinct molecular roles in the detection of glycerol and FAs.

It remains unclear whether *Gr64e* alone is sufficient for glycerol detection. Ectopic expression of *Gr64e* in olfactory receptor neurons confers glycerol responses [27], but *Gr64e* requires *Gr64b* as a co-receptor to confer glycerol responses on sweet GRNs [36]. To address this ambiguity, we used *Gr5a*-*GAL4* or *Ir76b*-*GAL4*, which labels low-salt sensing GRNs [20], to misexpress *Gr64b* alone, *Gr64e* alone, or *Gr64b* and *Gr64e* together in sweet GRNs or low-salt sensing GRNs of *Gr64af* flies, respectively. The misexpression of *Gr64b* and *Gr64e* together confers glycerol sensitivity in both sweet GRNs and low-salt sensing GRNs of *Gr64af* flies (Fig 4D–4G). Co-expression of *Gr64b* and *Gr64e* together in sweet GRNs of *Gr64af* flies restores their PER responses to glycerol (Fig 4H). In addition, introduction of *Gr64b* and *Gr64e* in bitter GRNs of *Gr64af* flies under the control of *Gr66a*-*GAL4*, which labels bitter GRNs [34], confers glycerol response (S3 Fig). These data suggest glycerol detection occurs through the direct activation of heteromeric ion channels formed by *Gr64b* and *Gr64e*.

**Fig 5:**
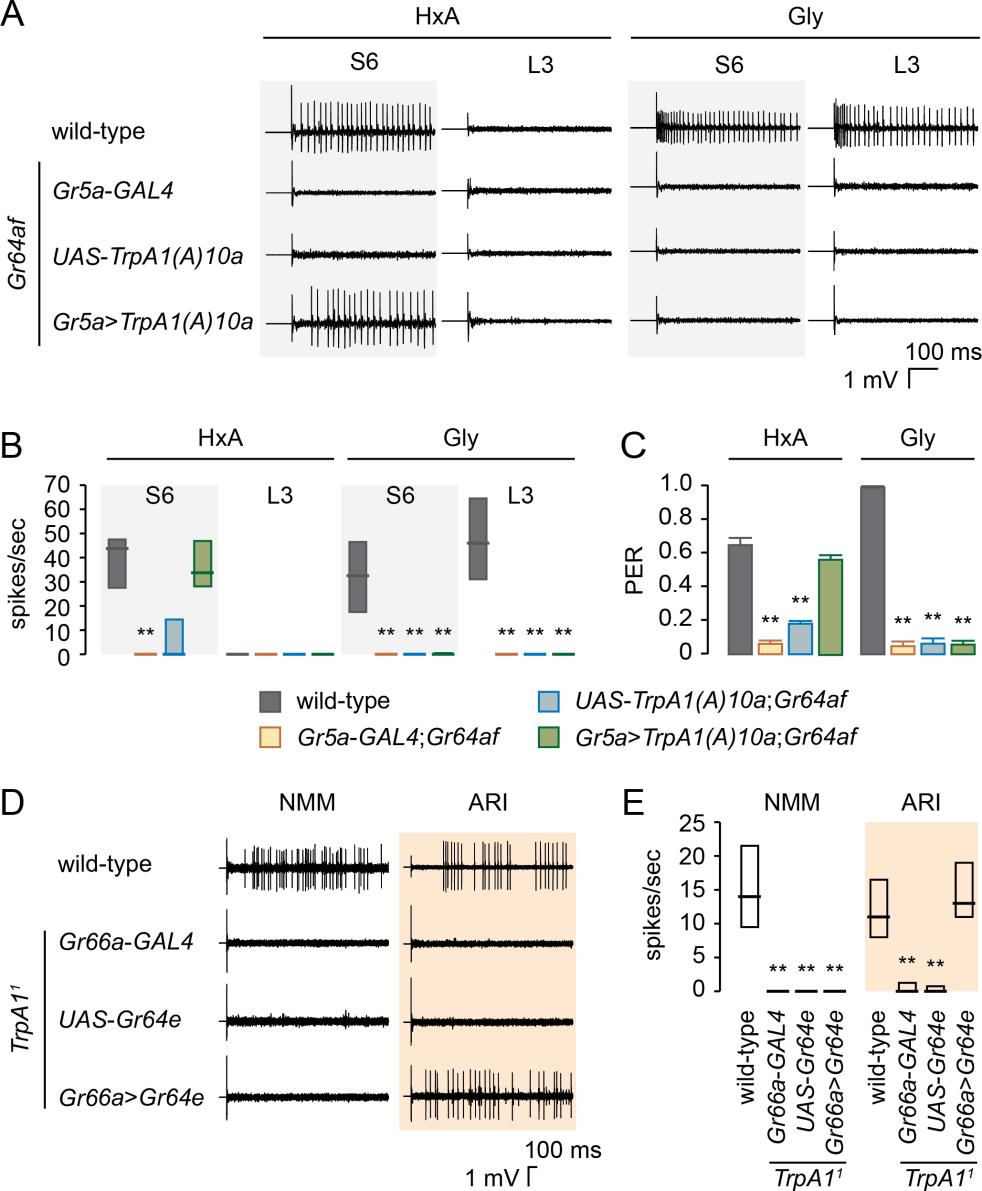
Functional redundancy between GR64e and TRPA1 downstream of PLC. (A and B) Representative traces (A) and response frequencies (B) from S6 and L3 sensilla of *Gr64af* flies expressing *TrpA1(A)10a* in sweet GRNs, as evoked by 1% HxA and 10% glycerol. n = 5–10. **p < 0.001 (Kruskal-Wallis with Mann-Whitney *U post-hoc* tests). (C) PER analysis to determine whether expression of *TrpA1(A)10a* under the control of *Gr5a*-*GAL4* rescues the *Gr64af* defect in FA sensing. Solutions of 0.4% HxA and 5% glycerol were used. n = 5–11. **p < 0.001 (one-way ANOVA with *post*-*hoc* Tukey tests). (D and E) Representative traces (D) and response frequencies (E) of S2 sensilla responding to 1 mM NMM and S6 sensilla responding to 1 mM ARI, all from *TrpA1*^*1*^ mutant flies expressing *Gr64e*. n = 8–21. **p < 0.001 (Kruskal-Wallis with Mann-Whitney *U post-hoc* tests). Data are presented as medians and quartiles (B and E) or as means ± SEM (C).

Although both *Gr64e* and PLC are required for FA detection in sweet GRNs, it is unclear how they function together. It is possible that *Gr64e* acts as a GPCR that detects HxA and functions upstream of PLC. This is unlikely, however, because sweet GRNs of L-type sensilla expressing *Gr64e* do not respond to HxA. To exclude the possibility that sweet GRNs of L-type sensilla lack other factors required for PLC signaling, we used *Gr5a*-*GAL4* to express either *Gαq*/*norpA* or *Gr64e*/*Gαq*/*norpA* in sweet GRNs. Neither of these combinations confers HxA responsiveness on the sweet GRNs of L-type sensilla (S4 Fig). A second hypothesis relating the function of *Gr64e* to PLC is that *Gr64e* functions downstream of PLC. *Drosophila trpA1* is expressed in a subset of bitter GRNs and required for avoidance to NMM [25], a tissue damaging reactive electrophile and ARI [23], a plant drived antifeedant. TRPA1 can be activated directly by NMM[25] and has also been associated with PLC signaling in ARI avoidance [23]. We hypothesize that if both TRPA1 and GR64e function downstream of PLC, TRPA1 and GR64e should be able to substitute for one another with regard to PLC signaling. We misexpressed either the thermosensory isoform *TrpA1(B)* or the chemosensory isoform *TrpA1(A)* in sweet GRNs of *Gr64af* flies to explore whether TRPA1 can replace the function of GR64e in FA sensing but not glycerol detection. We found *TrpA1* expression in sweet GRNs of *Gr64af* flies rescues HxA-evoked electrophysiological responses in their S-type sensilla and their HxA-evoked PER responses (Fig 5A–5C, S5 Fig). It does not, however, rescue glycerol detection. Furthermore, we also confirmed that functional replacement of GR64e with TRPA1 was dependent on PLC. Expression of *TrpA1* or *Gr64e* in sweet GRNs of *norpA*^*P24*^,*Gr64af* double mutant flies does not restore the response to HxA (S6 Fig).

We next asked whether GR64e can replace the function of TRPA1 in sensing noxious chemicals. We found that ARI elicits similar electrophysiological responses in wild-type and *TrpA1*^*1*^ flies expressing *Gr64e* in their bitter GRNs (Fig 5D and 5E). *TrpA1*^*1*^ flies expressing *Gr64e* in bitter GRNs do not, however, respond to NMM, a direct TRPA1 activator. These data further support *Gr64e* acts downstream of PLC for FA detection.

## Discussion

Here, we show that *Gr64e*—a sweet clade *Gr* required for glycerol detection [22]—is also essential for the gustatory detection of FAs. Although *Gr64e* is required in sweet GRNs for the detection of both glycerol and FAs, the molecular mechanisms by which it does so are different.

**Fig 6:**
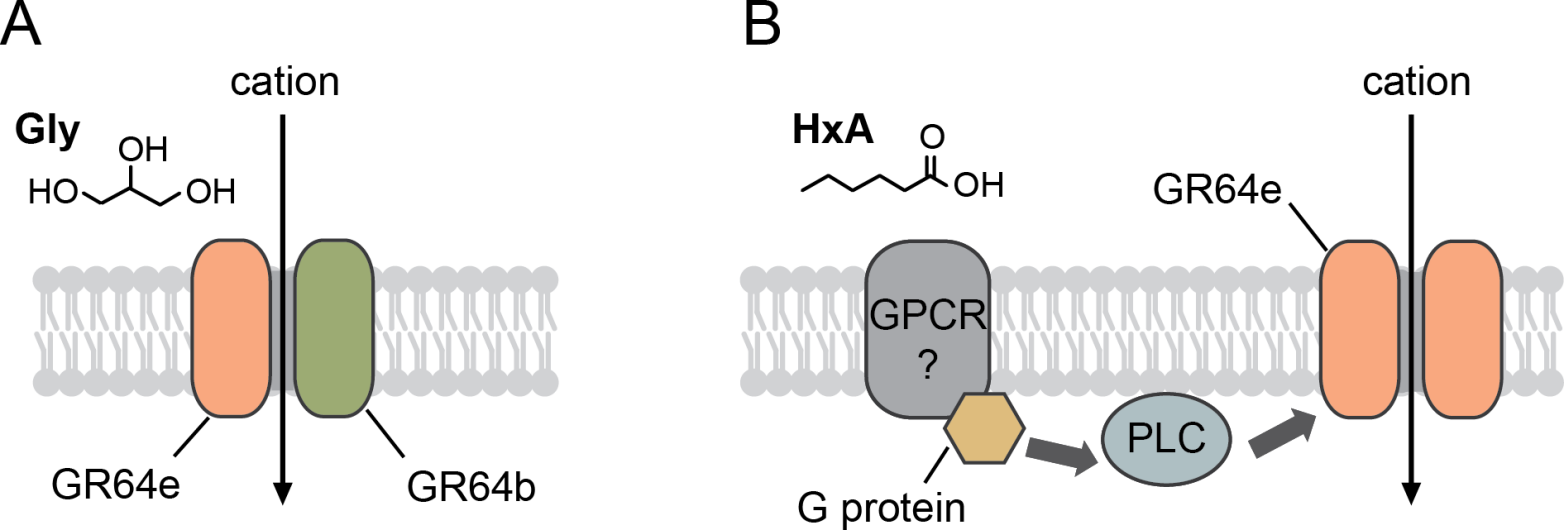
Models for activation of GR64e in fatty acid sensing and glycerol sensing. (A) Schematic model for GR64b and GR64e functioning as a ligand-gated channel in glycerol sensing. (B) Model for activation of GR64e in FA sensing. Activation of an unknown FA receptor stimulates phospholipase C (PLC), thereby activating GR64e.

Glycerol evokes action potentials in sweet GRNs in L-, I-, and S-type sensilla in a PLC-independent manner (Fig 4A and 4B) [22]. Freeman et al. reported that single sweet GRs alone confer the responses to various sugars including glycerol when they mis-express them in olfactory neurons [27]. Only the combination of *Gr64b* and *Gr64e*, however, confers glycerol responsiveness on the sweet GRNs [36], low-salt sensing GRNs, and bitter GRNs of *Gr64af* flies. This suggests *Drosophila* GRs form heteromeric complexes for sensing sugars. Since *Gr64b*/*Gr64e*-misexpressing low-salt sensing GRNs or bitter GRNs produce fewer glycerol-evoked action potentials than sweet GRNs, we speculate that there are unknown additional *Grs* in sweet GRNs that facilitate the formation of high affinity glycerol receptors. This would be similar to our findings with the L-canavanine receptor [15]. Based on the characterization of GRs for bitter sensing [15, 37], the detection of glycerol occurs through the direct activation of ion channels formed by *Gr64b* and *Gr64e* (Fig 6A), but it remains unclear whether unknown intracellular signaling components also contribute to the function of sweet GRs.

FAs selectively activate sweet GRNs in S-type sensilla in a PLC-dependent manner. Of the nine sweet clade *Grs* (i.e., *Gr5a*, *Gr43a*, *Gr61a*, and *Gr64a-f*), only *Gr64e* is required for FA detection. *Gr64e* seems unlikely to be a FA receptor for several reasons. First, the sweet GRNs in L- and I- type sensilla, where endogenous *Gr64e* is expressed [28], respond only to glycerol, not FAs (Fig 3). Second, overexpression of G-protein signaling components (*Gαq* and *norpA*) alone or together with *Gr64e* (*Gr64e*, *Gαq*, and *norpA*) in sweet GRNs of L-type sensilla does not endow FA sensitivity (S4 Fig). Finally, although there are reports that the distantly related olfactory receptors function as both GPCRs and ionotropic receptors [38, 39], the inverse topology of GRs relative to GPCRs is further evidence that *Gr64e* is unlikely a direct FA receptor. We were unable to exclude the possibility that *Gr64e* acts as an accessory protein for an unknown FA-responsive GPCR or the possibility that the absence of other accessory proteins (i.e., CD36 [40]) in sweet GRNs of L-type sensilla explains their inability to respond to HxA. Furthermore, the functional redundancy we identified between GR64e and TRPA1 in PLC-specific functions (e.g., FA but not glycerol detection by GR64e and ARI but not NMM detection by TRPA1) suggests *Gr64e* functions downstream of PLC (Fig 6B). Although GR64e and TRPA1 are functionally interchangeable downstream of PLC, it remains unclear whether they share the same molecular mechanism of activation. GR64e can be activated by hydrolysis of phosphoinositide by PLC, elevation of intracellular calcium, or diacylglycerol. Alternatively, Gr64e may be a voltage-gated channel that is not directly coupled to the PLC pathway.

Two *Drosophila* species, *D*. *psedoobscura* and *D*. *persimilis* carry pseudogenized versions of *Gr64e* and do not respond to glycerol [22]. If these two species have also lost gustatory sensitivity to FAs, it will confirm the evolutionary conservation of this dual function for *Grs*.

Because this is the first time a *Drosophila* GR has been found to function downstream of PLC, our results extend the molecular repertoire of the GR family of proteins. This is particularly intriguing because there are *Grs* expressed in the antenna [28, 41] and in the enteroendocrine cells of the gut [42]. Rather than acting in the direct detection of ligands in these non-gustatory cells, these GRs may mediate novel sensory modalities via distinct molecular mechanisms.

FAs act as sources of energy, but also as structural components of membranes. In addition, they have multiple biological roles in metabolism, cell division, and inflammation [43]. In flies, changes in the FA composition of membranes via FA deprivation influences cold tolerance and synaptic function [44, 45]. Dietary FAs also modulate mitochondrial function and longevity [46]. Thus, animals must ingest dietary FAs for survival. Indeed, regular laboratory *Drosophila* foods also contain FAs [45]. It is unsurprising that FA taste is well-conserved between mammals and flies, which are required for PLC pathway in contrast to other taste modalities in flies. Since GPR40 and GPR120 are strong FA receptor candidates in mammals [5], an FA-sensitive GPCR may also be selectively expressed in the sweet GRNs of S-type sensilla in flies. It will be interesting to determine whether the *Drosophila* orthologue of the mammalian FA receptor or any other GPCRs are involved in FA detection.

## Materials and methods

### Fly stocks

Flies were maintained on cornmeal-molasses-yeast medium at 25°C and 60% humidity with a 12h/12h light/dark cycle. The fly medium recipe is based on the Bloomington recipe (https://bdsc.indiana.edu/information/recipes/molassesfood.html) and composed of 3% yeast (SAF Instant Yeast), 6% cornmeal (DFC-30102, Hansol Tech, Korea), 8% molasses (extra fancy Barbados molasses, food grade, Crosby Molasses Co., Ltd. of Canada), and 1% agar (DFA-30301, Hansol Tech) for the nutrients and the hardener. It also includes 0.8% Methyl 4-hydroxybenzoate (H5501, Sigma-Aldrich, Saint Louis, MO), 0.24% propionic acid (P1386, Sigma-Aldrich), and 0.0028% phosphoric acid (695017, Sigma-Aldrich) as preservatives. For optogenetic experiments, instant fly food was purchased from Carolina (Burlington, NC, #173200). *Gr64d*^*1*^ was described previously [47]. *Gr5a-GAL4*, *Gr66a-GAL4*, *Gr43a*^*GAL4*^, *Gr5a*^*LEXA*^, *Gr64a*^*GAL4*^, *Gr64b*^*LEXA*^, *Gr64c*^*LEXA*^, *Gr64e*^*LEXA*^, and *Gr64f*^*LEXA*^ were provided by H. Amrein. *ΔGr64a*^*1*^, *ΔGr64a*^*2*^, and *ΔGr61a*^*1*^ were provided by J. Carlson. *UAS-Gr64b*, *UAS-Gr64c*, and *UAS-Gr64e* were provided by A. Dahanukar. *Gr64*^*ab*^, *Ir76b-GAL4*, and *TrpA1*^*1*^ were provided by C. Montell, *UAS-TrpA1(A)10a*, *UAS-TrpA1(A)10b*, and *UAS-TrpA1(B)10a* were provided by P. Garrity, and *LexAop-Kir2*.*1* was provided from B. Dickson, respectively. *UAS*-*ReaChR* (BL53741), *norpA*^*P24*^ (BL9048), *UAS*-*norpA* (BL26273), *UAS*-*Gαq* (BL30734), *Gr64e-GAL4* (BL57667), and *UAS-Kir2*.*1* (BL6595) were obtained from the Bloomington Stock Center. *nos*-*Cas9* (#CAS-0001) was obtained from NIG-FLY. All the mutant lines and transgenic lines were backcrossed for five generations to the *w*^*1118*^ control genotype. For clarity, the *w*^*1118*^ line is referred to as wild-type throughout the manuscript.

### Generation of *Gr64af* mutant

We used CRISPR/Cas9 system to generate *Gr64af* flies [48]. We selected two target sites for deletion of the whole *Gr64* cluster using DRSC Find CRISPRs (http://www.flyrnai.org/crispr) and CRISPR optimal target finder (http://tools.flycrispr.molbio.wisc.edu/targetFinder): one near the 5’ end of *Gr64a* (GAATCCTCAACAAACTTCGGTGG, the Protospacer Adjacent Motif is underlined) and one near the 3’ end of *Gr64f* (GGTCGTTGTCCTCATGAAATTGG). We synthesized oligomers and cloned them into the BbsI site on pU6-BbsI-ChiRNA (Addgene #45946). After injecting two pU6-ChiRNA targeting constructs into *nos*-*Cas9* embryos at 500 ng/μl each, we screened the resulting flies for deletions via PCR of genomic DNA isolated from the G_0_ generation. The primers we used for deletion confirmation were as follows: TCTCGGCAGCTAATCGAAAT and GCGACCATTCTTTGTGGAAT.

### Proboscis extension reflex (PER) assay

We collected 3–5-day-old flies in fresh food for 24 hours. Then, we starved them for 18 hours in vials containing 1% agarose. After anaesthetizing the flies on ice, we mounted them on slide glasses with melted 1-tetradecanol (185388, Sigma-Aldrich). We then allowed the flies to recover for 1–2 hours and ensured they were satiated with water before the assay. For each test solution, we used a 1 ml syringe with a 32-gauge needle to apply a single droplet directly to the labellum. We dissolved FAs in 4% ethanol. Each experimental group contained 24 flies, half were mated males and half were mated females, attached to a slide glass. All PER experiments were performed at the same time to eliminate any circadian effects. We report PER responses as the number of responding flies/total flies.

### Tip recording

We performed tip recordings as previously described [49, 50]. Briefly, we immobilized 5–7-day-old flies by inserting a reference electrode—a glass capillary filled with Ringer’s solution—through the thorax and into the labellum. Then, we stimulated the indicated labellar sensilla with a recording electrode (10–20 μm tip diameter) containing test chemicals in 30 mM tricholine citrate (TCC) as the electrolyte. After connecting the recording electrode to a 10X preamplifier (TastePROBE; Syntech, Hilversum, The Netherlands), we recorded action potentials at 12 kHz with a 100–3,000 Hz band-pass filter using a data acquisition controller (Syntech), sorted the spikes based on amplitude, and analyzed them with the Autospike 3.1 software package (Syntech).

### Chemicals

We purchased hexanoic acids (153745), octanoic acids (2875), oleic acids (01008), linoleic acids (L1376), sucrose (S9378), α-D-glucose (158968), D-(-)-fructose (F3510), D-(+)-trehalose dihydrate (90210), glycerol (G9012), *N*-methylmaleimide (389412), aristolochic acid I (A5512), and tricholine citrate (T0252) from Sigma-Aldrich. Sodium chloride (S0520) was purchased from Duchefa Biochemie (Haarlem, Netherland).

### Optogenetics

3–4-day-old flies were transferred to vials containing instant *Drosophila* medium with or without 400 μM all trans-retinal (R2500, Sigma-Aldrich), respectively. After feeding the flies retinal for a week, they were mounted into 200 μl pipette tips. Then, they were exposed to LED light (wavelength of 627 nm). PER responses were monitored by video camera and counted manually.

### Statistics

We performed all statistical analyses using SPSS Statistics 23 (IBM Corporation, Armonk, NY). We tested normality and homoscedasticity using the Kolmogorov-Smirnov and Levene tests. PER responses are displayed as means ± SEM. We used unpaired Student’s t-tests or one-way ANOVAs with Tukey *post-hoc* tests to analyze the PER data. All electrophysiological data are presented as medians with quartiles. We used the Mann-Whitney *U*-test or Kruskal-Wallis test with Mann-Whitney *U post-hoc* tests to determine whether the medians for each genotype were significantly different.

## Supporting information

S1 Fig Schematic showing the individual *Gr64* cluster gene mutants. The deletions and insertions of specific coding sequences (i.e., *GAL4* or *LEXA*) are indicated.
(TIF)

S2 Fig Labellar PER responses to HxA in *Gr64e*-expressing GRNs silenced flies. (A) PER responses to 0.4% HxA and 5% Gly in control flies (*UAS-Kir2*.*1*/+) and in flies expressing the inwardly rectifying potassium channel Kir2.1 under the control of *Gr64e*-*GAL4* (genotype: *Gr64e*-*GAL4*/+;*UAS*-*Kir2*.*1*/+). n = 3. **p < 0.001 (unpaired Student’s *t*-test). (B) PER responses to 0.4% HxA and 5% Gly in control flies (*Gr64e*^*LEXA*^/+) and flies expressing the inwardly rectifying potassium channel Kir2.1 under the control of *Gr64e*^*LEXA*^ (genotype: *LexAop*-*Kir2*.*1*/+;*Gr64e*^*LEXA*^/+). n = 3–5. **p < 0.001 (unpaired Student’s *t*-test).
(TIF)

S3 Fig Co-expression of *Gr64b* and *Gr64e* in bitter GRNs confers glycerol responsiveness. Representative traces (A) and response frequencies (B) elicited by 10% glycerol from S6 sensilla in *Gr64af* flies expressing *Gr64b* and *Gr64e* under the control of *Gr66a*-*GAL4*. n = 5–6. *p < 0.01, **p < 0.001 (Kruskal-Wallis with Mann-Whitney *U post-hoc* tests).
(TIF)

S4 Fig Electrophysiological responses to HxA after ectopic expression of PLC signaling components. Representative traces (A) and response frequencies (B) evoked by 1% HxA from L-type sensilla expressing *Gαq* and *norpA* in sweet GRNs under the control of *Gr5a*-*GAL4*. n = 5–8.
(TIF)

S5 Fig Ectopic expression of *TrpA1* in sweet GRNs of *Gr64af* flies rescues their responses to HxA but not glycerol. Representative traces (A) and response frequencies (B) from S6 and L3 sensilla of the indicated genotypes elicited by 1% HxA and 10% glycerol solutions. n = 5–10. **p < 0.001 (Kruskal-Wallis with Mann-Whitney *U post-hoc* tests).
(TIF)

S6 Fig HxA responses in sweet GRNs of S-type sensilla require *norpA*. Representative traces (A) and response frequencies (B) to 1% HxA in S6 sensilla of the indicated genotypes. n = 4–6. **p < 0.001 (Kruskal-Wallis with Mann-Whitney *U post-hoc* tests).
(TIF)
