# Supplementary figures and images for: Drosophila Gr64e mediates fatty acid sensing via the phospholipase C pathway
Source: PLoS Genet. 2018 Feb 8;14(2):e1007229. doi: 10.1371/journal.pgen.1007229 (PMC5821400; doi:10.1371/journal.pgen.1007229)

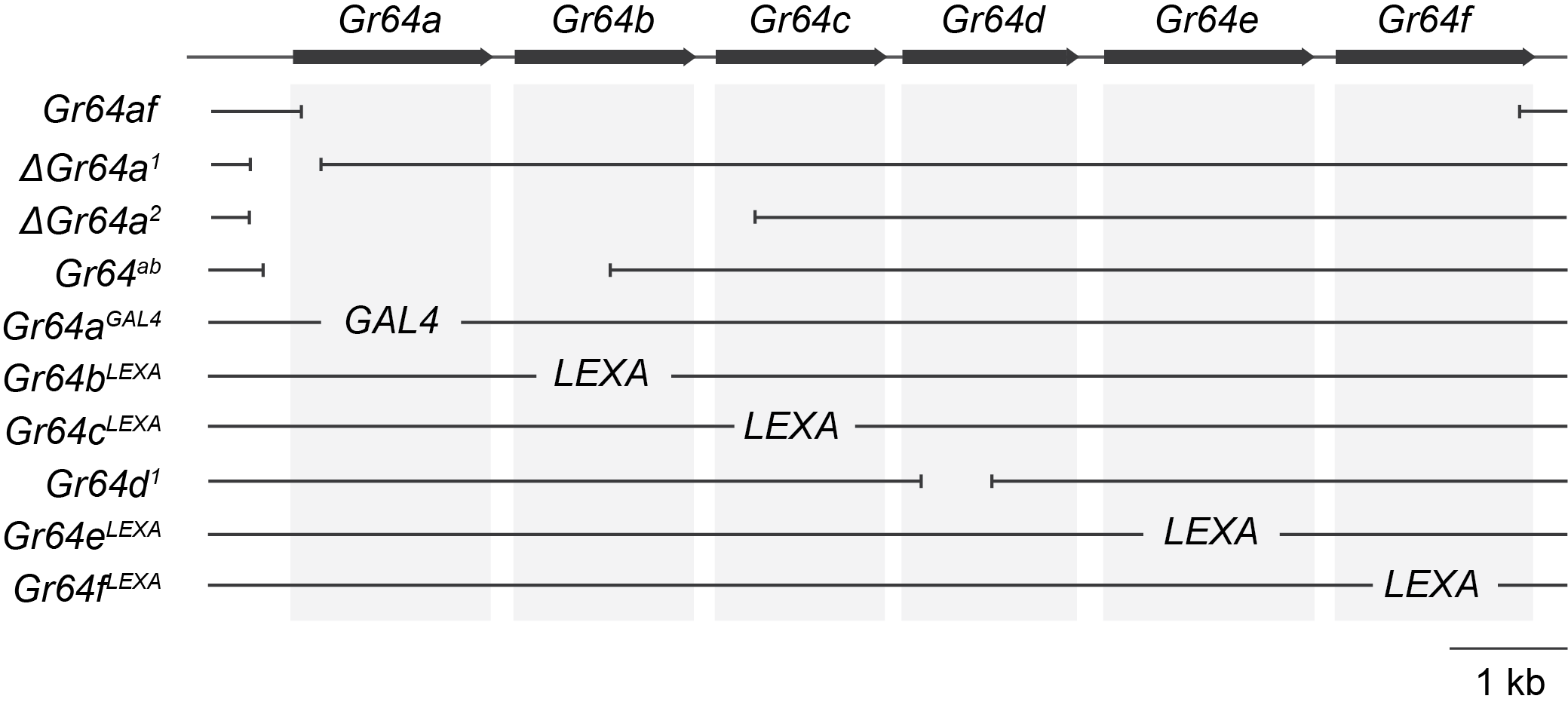

Supplement: S1 Fig — The deletions and insertions of specific coding sequences (i.e., GAL4 or LEXA) are indicated. (TIF) [file pgen.1007229.s001.tif]

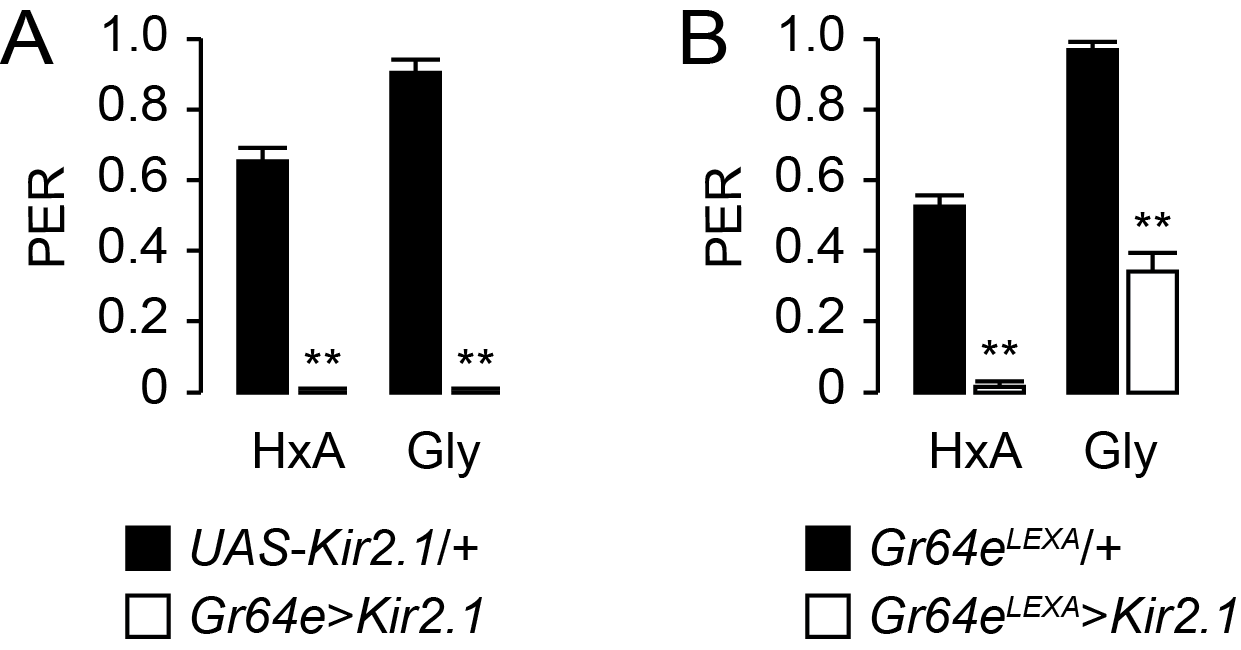

Supplement: S2 Fig — (A) PER responses to 0.4% HxA and 5% Gly in control flies (UAS-Kir2.1/+) and in flies expressing the inwardly rectifying potassium channel Kir2.1 under the control of Gr64e-GAL4 (genotype: Gr64e-GAL4/+;UAS-Kir2.1/+). n = 3. **p < 0.001 (unpaired Student’s t-test). (B) PER responses to 0.4% HxA and 5% Gly in control flies (Gr64eLEXA/+) and flies expressing the inwardly rectifying potassium channel Kir2.1 under the control of Gr64eLEXA (genotype: LexAop-Kir2.1/+;Gr64eLEXA/+). n = 3–5. **p < 0.001 (unpaired Student’s t-test). (TIF) [file pgen.1007229.s002.tif]

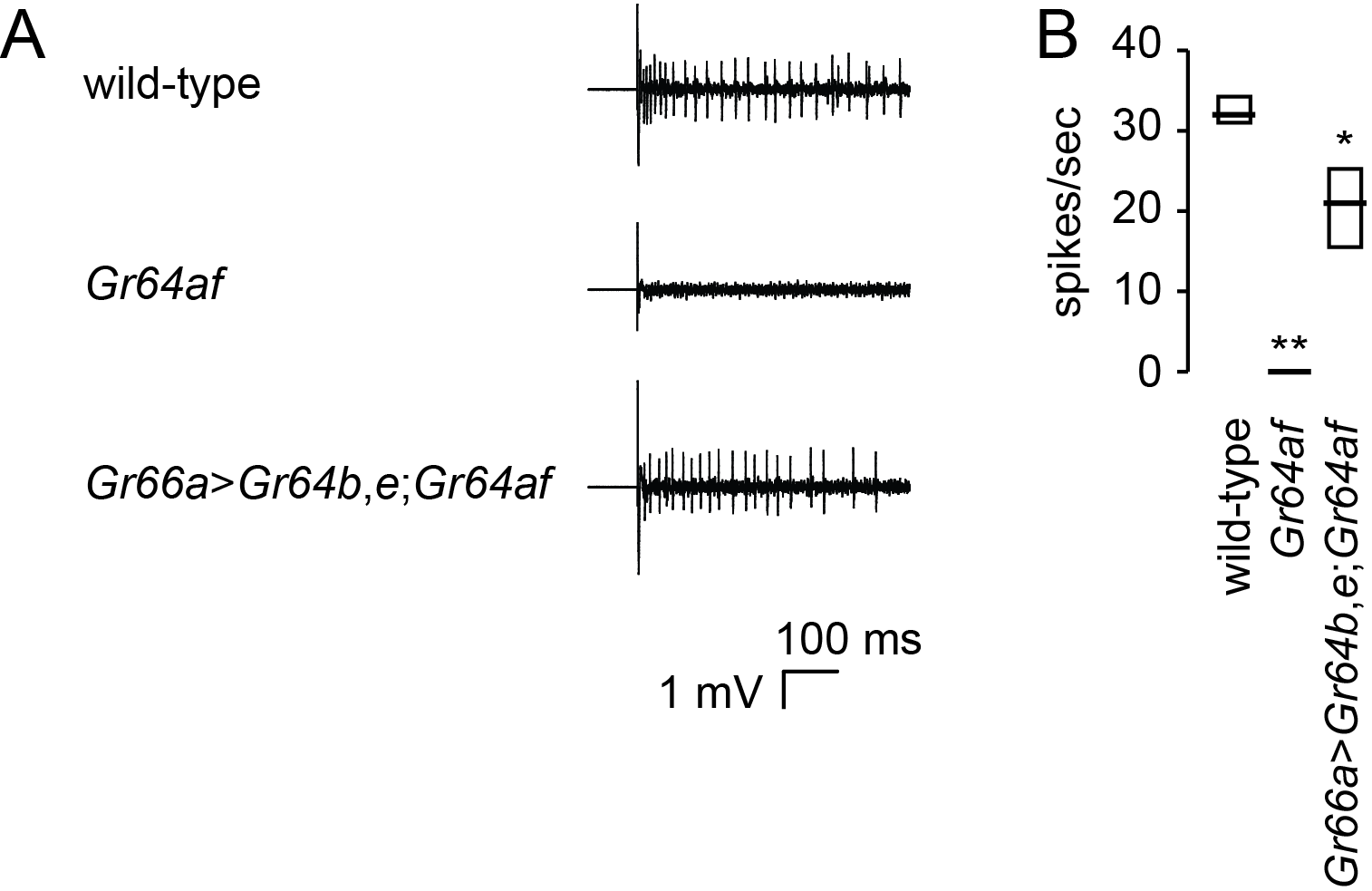

Supplement: S3 Fig — Representative traces (A) and response frequencies (B) elicited by 10% glycerol from S6 sensilla in Gr64af flies expressing Gr64b and Gr64e under the control of Gr66a-GAL4. n = 5–6. *p < 0.01, **p < 0.001 (Kruskal-Wallis with Mann-Whitney U post-hoc tests). (TIF) [file pgen.1007229.s003.tif]

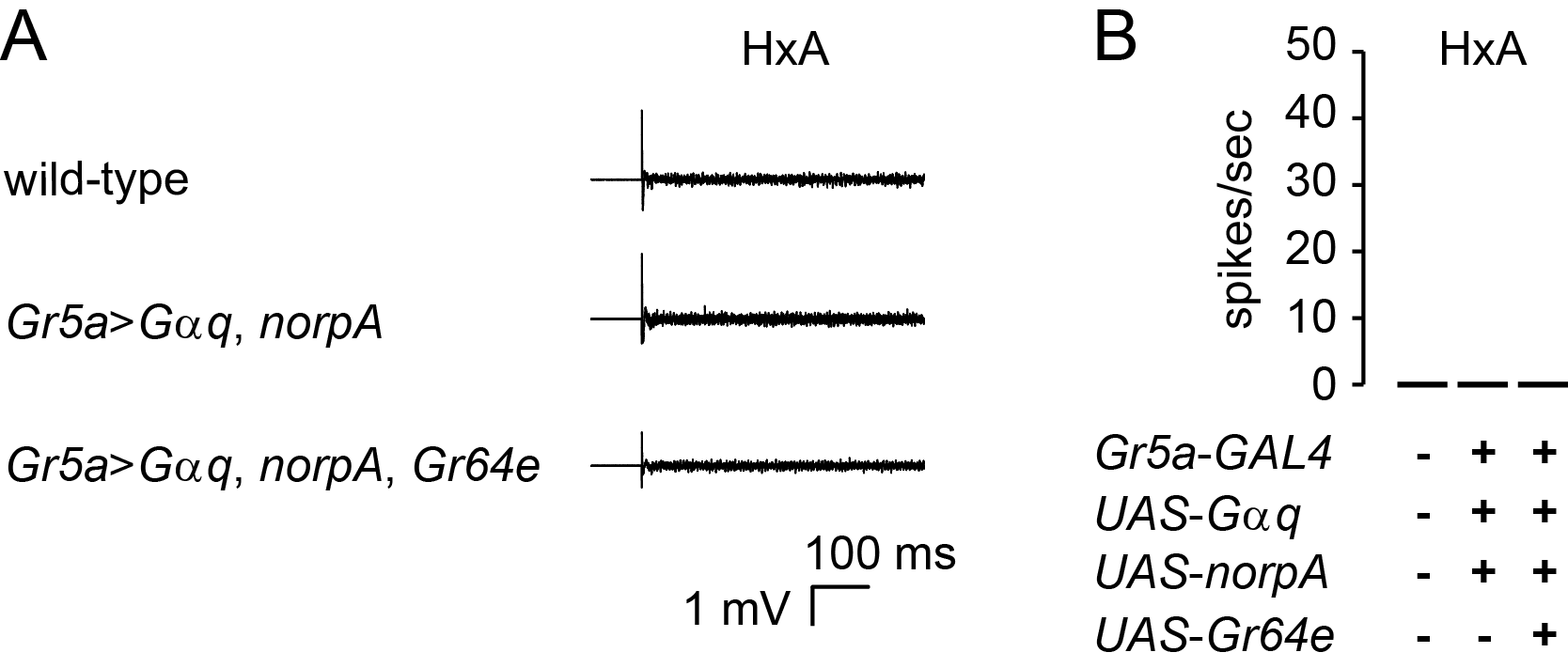

Supplement: S4 Fig — Representative traces (A) and response frequencies (B) evoked by 1% HxA from L-type sensilla expressing Gαq and norpA in sweet GRNs under the control of Gr5a-GAL4. n = 5–8. (TIF) [file pgen.1007229.s004.tif]

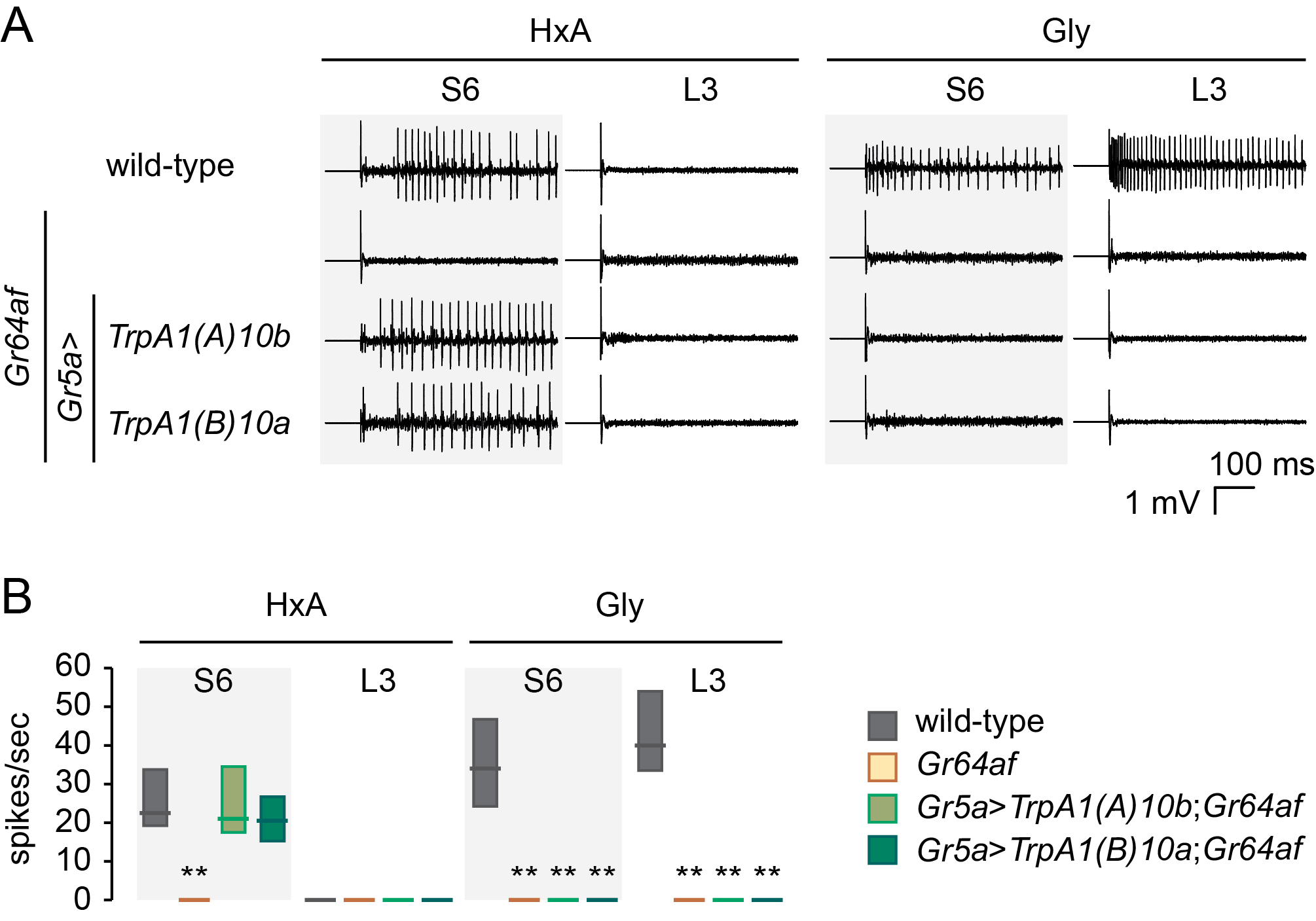

Supplement: S5 Fig — Representative traces (A) and response frequencies (B) from S6 and L3 sensilla of the indicated genotypes elicited by 1% HxA and 10% glycerol solutions. n = 5–10. **p < 0.001 (Kruskal-Wallis with Mann-Whitney U post-hoc tests). (TIF) [file pgen.1007229.s005.tif]

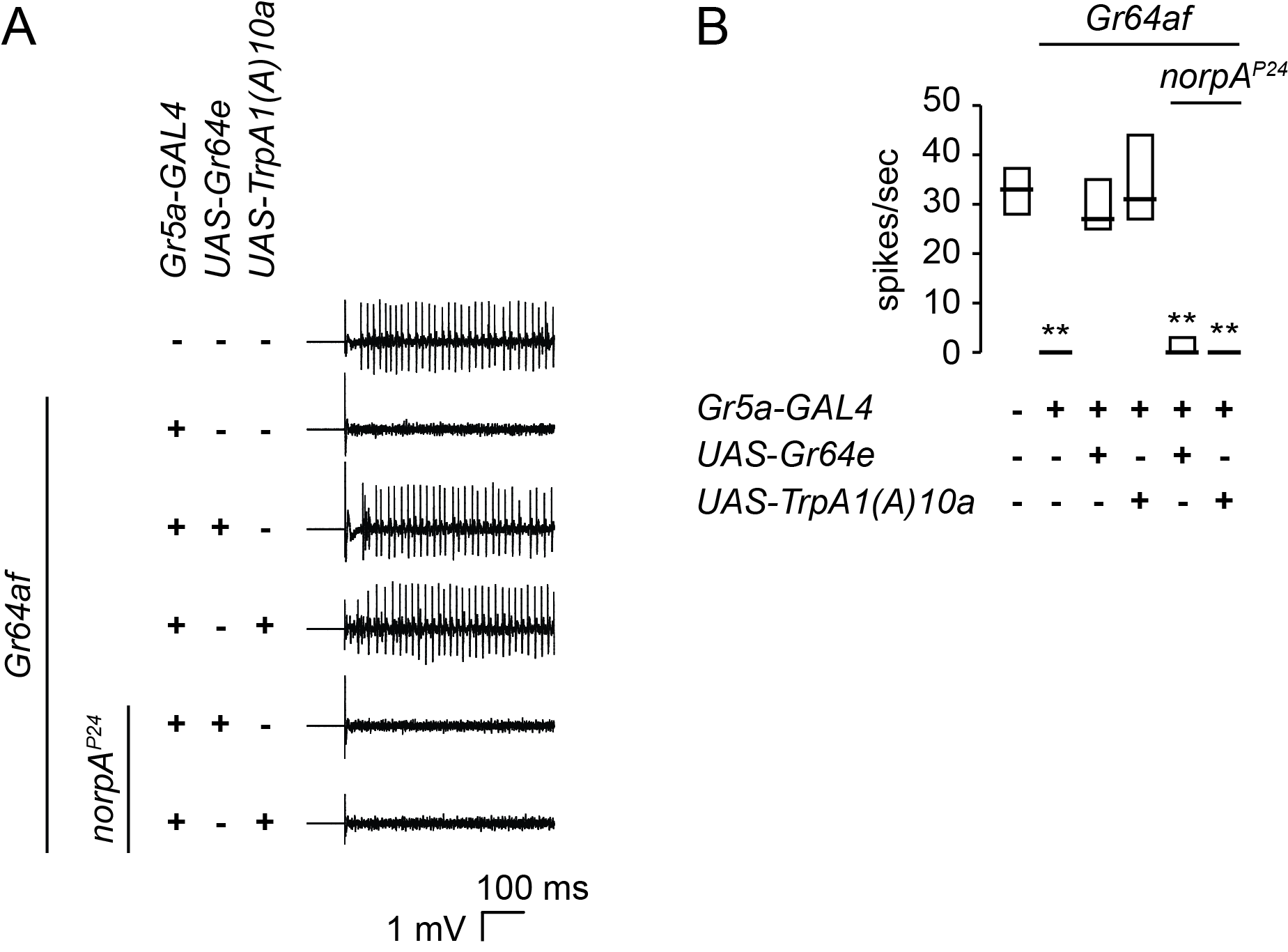

Supplement: S6 Fig — Representative traces (A) and response frequencies (B) to 1% HxA in S6 sensilla of the indicated genotypes. n = 4–6. **p < 0.001 (Kruskal-Wallis with Mann-Whitney U post-hoc tests). (TIF) [file pgen.1007229.s006.tif]
